# Sequence–Activity Relationship of Angiotensin-Converting Enzyme Inhibitory Peptides Derived from Food Proteins, Based on a New Deep Learning Model

**DOI:** 10.3390/foods13223550

**Published:** 2024-11-07

**Authors:** Dongya Qin, Xiao Liang, Linna Jiao, Ruihong Wang, Yi Zhao, Wenjun Xue, Jinhong Wang, Guizhao Liang

**Affiliations:** Key Laboratory of Biorheological Science and Technology, Ministry of Education, College of Bioengineering, Chongqing University, Chongqing 400044, China; qindongya@jflab.ac.cn (D.Q.); 20241901014@stu.cqu.edu.cn (X.L.); lnjiao@cqu.edu.cn (L.J.); wrh5742@163.com (R.W.); 20221901021@stu.cqu.edu.cn (Y.Z.); xwj15566509785@163.com (W.X.); 202319131086t@stu.cqu.edu.cn (J.W.)

**Keywords:** angiotensin-converting enzyme inhibitory peptide (ACEiP), food-derived peptide, deep learning, amino acid descriptor

## Abstract

Food-derived peptides are usually safe natural drug candidates that can potentially inhibit the angiotensin-converting enzyme (ACE). The wet experiments used to identify ACE inhibitory peptides (ACEiPs) are time-consuming and costly, making it important and urgent to reduce the scope of experimental validation through bioinformatics methods. Here, we construct an ACE inhibitory peptide predictor (ACEiPP) using optimized amino acid descriptors (AADs) and long- and short-term memory neural networks. Our results show that combined-AAD models exhibit more efficient feature transformation ability than single-AAD models, especially the training model with the optimal descriptors as the feature inputs, which exhibits the highest predictive ability in the independent test (Acc = 0.9479 and AUC = 0.9876), with a significant performance improvement compared to the existing three predictors. The model can effectively characterize the structure–activity relationship of ACEiPs. By combining the model with database mining, we used ACEiPP to screen four ACEiPs with multiple reported functions. We also used ACEiPP to predict peptides from 21,249 food-derived proteins in the Database of Food-derived Bioactive Peptides (DFBP) and construct a library of potential ACEiPs to facilitate the discovery of new anti-ACE peptides.

## 1. Introduction

ACE-inhibitory peptides (ACEiPs) are short peptides with about 2–19 amino acid residues. They can block the conversion of angiotensin I to angiotensin II by inhibiting ACE in the renin-angiotensin-aldosterone system, leading to the suppression of vasoconstriction and a reduction in blood pressure [1,2]. Many ACEiPs have been identified from milk, animal, plant, seafood, and microorganism sources by fermentation, enzymatic hydrolysis, and synthetic methods [3,4,5,6,7,8]. These ACEiPs can inhibit ACE in vitro and even exhibit similar or better blood-pressure-lowering effects than *Captopril* (an ACE inhibitor for the treatment of hypertension) in animal experiments [9,10,11]. More importantly, some ACEiPs exhibit multiple activities (antihypertensive, antioxidant, antidiabetic, anticancer, etc.) according to the Database of Food-derived Bioactive Peptides (DFBP) [12]. Therefore, ACEiPs have the advantages of high activity, multiple functions, multiple sources, and easy access, especially food-derived multifunctional peptides with ACE-inhibitory activity, which are expected to become candidates for replacing synthetic drugs in the treatment of cardiovascular diseases [2,7,13,14].

The peptide library, consisting of 20 natural amino acids, is extensive, so the experimental identification of bioactive peptides (including ACEiPs) is labor-intensive, time-consuming, and costly [15]. Recently, computational methods have emerged as a new strategy for the large-scale screening of bioactive peptides, including quantitative structure−activity relationship (QSAR), machine learning (ML), and deep learning (DL) techniques [16,17]. In QSAR studies, amino acid descriptors (AADs) are commonly used to characterize the sequence/structure characteristics of peptides [16]. Multiple AADs have been proposed to characterize ACEiPs, such as z-scales [18], HESE [19], and FASGAI [20], showing favorable interpretability regarding the structure–activity relationship of peptides. ML-based methods have shown advantages in the large-scale prediction of peptides by using physicochemical parameters, structural properties, and experimental data as feature inputs to train models and mine the bio-information found in the multi-dimensional data [15]. AHTpin [21] and mAHTPred [22] are currently the only two accessible predictors for antihypertensive peptides (AHTPs). AHTpin uses amino acid and atomic composition features to develop multiple classification models for small peptides, medium peptides, and large peptides, among which the model has achieved the highest accuracy of 84.21% for large peptides. mAHTPred is a state-of-the-art AHTP classifier that combines an extremely randomized tree and 51 feature descriptors derived from eight different feature encodings, achieving the highest accuracy of 88.3% compared to six different ML algorithms. DL-based methods can use diverse input data, especially when processing images, and natural language to accurately capture the sequence/structure characteristics of bioactive peptides. With the exception of one off-line model (ProtBERT) [23], the DL technique has not yet been applied in the study of ACEiPs, but it has already been used in the study of several other bioactive peptides, such as sAMP-PFPDeep (an antibacterial peptide predictor based on a convolutional neural network) [24], iACP-DRLF (an anticancer peptide predictor based on long short-term memory (LSTM) neural networks and their derived networks) [25], and a multifunctional peptide predictor based on a convolutional neural network [26].

These computational methods have made important contributions to the screening of bioactive peptides. However, there is still insufficient research on the prediction of ACEiPs, and the possible reasons for this are as follows: (i) QSAR is currently used mainly for small-sample regression, while feature extraction is difficult when the lengths of ACEiPs are inconsistent; (ii) ML-based AHTP predictors can be used to screen ACEiPs, but the prediction accuracy (of less than 88.3% [22]) still needs to be improved, and the ML-based model usually requires a specific type and length of data as input parameters and is less interpretable; and (iii) no DL-based prediction server for ACEiPs is yet available. LSTM is a particular recurrent neural network for processing natural sequence data with different lengths, avoiding gradient disappearance and explosion problems during long-sequence training [27,28]. LSTM can memorize the important transfer information between sequences and forget non-important information by controlling the transmission state through three gated states (the input, forget, and output gates) [27]. Therefore, combining the learning ability of an LSTM neural network and the interpretability of AADs may be an efficient strategy to improve the prediction accuracy and explain the sequence/structure characteristics of ACEiPs.

**Figure 1:**
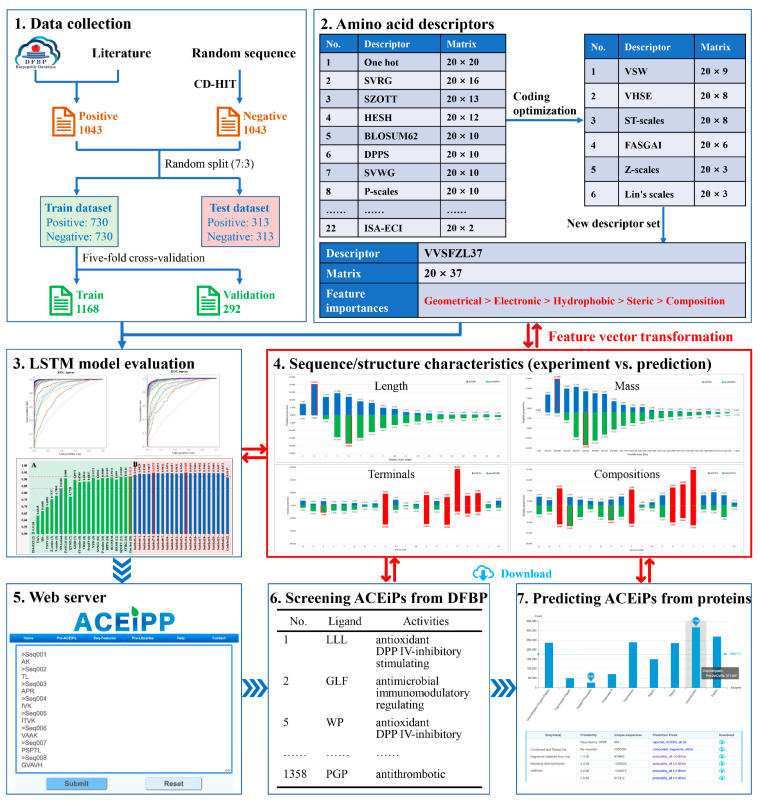
Workflow of ACEiPP: (**1**) collect the sequences of ACEiPs and non- ACEiPs; (**2**) translate the sequences into feature vectors by single and combined AADs; (**3**) train the LSTM models and learn the key features of peptides; (**4**) characterize the sequence/structure characteristics of peptides; (**5**) deploy the optimal model in the webserver to access the online prediction; (**6**) screen the ACEiPs with reported multiple activities from the DFBP [12]; and (**7**) predict the potential ACEiPs encoded in food-derived proteins.

To address the above challenges, we combined an LSTM neural network and AAD encodings to train an optimal model and explain the main sequence/structure characteristics of ACEiPs, thereby developing a predictor named ACEiPP (http://www.cqudfbp.net/ACEiPP/index.jsp, accessed on 4 November 2024) to predict potential ACEiPs (Figure 1). We used ACEiPP to screen four ACEiPs with multiple activities and constructed a library of 1,320,635 potential ACEiPs. To our knowledge, ACEiPP is the first DL-based online predictor for the high-efficiency prediction and screening of ACEiPs, reducing the significant time and resources required for early-stage screening and providing a strong foundation for subsequent experimental validation.

## 2. Materials and Methods

### 2.1. Benchmark and Independent Dataset

One benchmark dataset and two independent datasets were used for training the predictor and evaluating its prediction performance (Supplementary Table S1). After deleting reduplicate or controversial sequences with inconsistent experimental results, 1043 ACEiPs with IC_50_ values of below 1000 μM were selected as positive samples from three databases: the DFBP (http://www.cqudfbp.net/, accessed on 4 November 2024), BIOPEP-UWM (https://biochemia.uwm.edu.pl/biopep-uwm/, accessed on 4 November 2024) and AHTPDB (http://crdd.osdd.net/raghava/ahtpdb/, accessed on 4 November 2024), along with the related literature. It is worth mentioning that to ensure the model can effectively learn and extract the sequence features of ACE-inhibiting peptides, we ultimately chose 1000 μM as the sample classification threshold after numerous practical attempts and comprehensive consideration. This value allows us to maintain the reliability of the experimental data while avoiding the misclassification of peptides that still possess moderate inhibitory activity as being negative samples. To maintain the balance of the number and length distribution of positive and negative samples, a manually written Java program and CD-HIT [29] were used to generate random sequences and remove the sequences with a greater than 90% similarity with positive samples. In total, 1043 peptides were randomly selected as negative samples to balance the number of positive samples. Both positive and negative samples were split according to a 7:3 ratio to construct a benchmark dataset (benchmark_ACEiPs) and an independent dataset (independent_ACEiPs) [21]. The second independent dataset (saved as independent_AHTPs.txt) with a sequence length of ≥5 was downloaded from mAHTPred (http://thegleelab.org/mAHTPred/, accessed on 4 November 2024), and contained 386 AHTPs and 386 non-AHTPs [21].

### 2.2. Sequence Representation

In this step, 22 kinds of AADs were used to translate peptide sequences into feature vectors as inputs of neural networks, where One-hot and the other 21 kinds of descriptors described the types and physicochemical attributes of natural amino acids, respectively (Supplementary Table S2). Two encoding strategies were used, as follows: (i) single AADs were used to train the models using 22 independent AAD encoding matrices individually; and (ii) combined AADs were used to combine all the single AADs into a 20 × 149 matrix (CodeSet22), using “Leave-Group-Out” to sequentially delete single AADs from CodeSet22 to optimize the best combination coding.

### 2.3. LSTM Architecture

**Figure 2:**
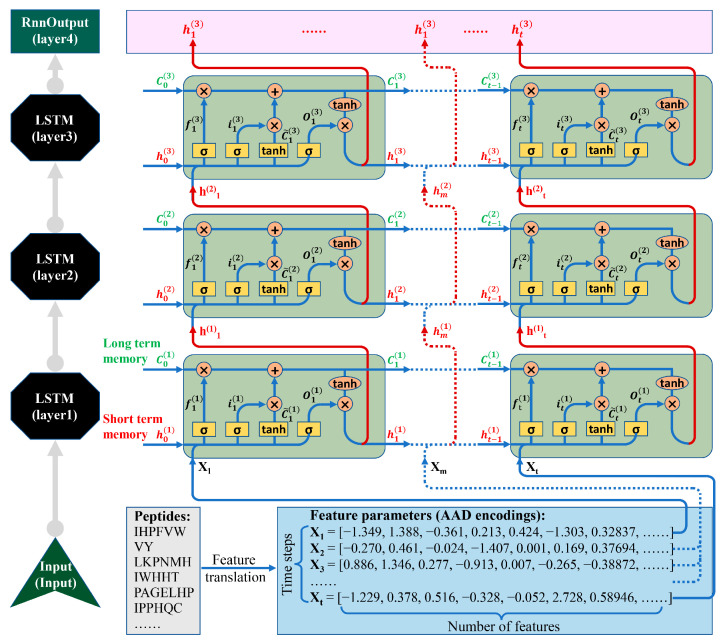
Flowchart of LSTM architecture. Four layers were set up, including three LSTM layers and one RnnOutput layer.

In this study, an LSTM neural network in a Deeplearning 4j (version 1.0.0-M1.1) framework (DL4J: https://deeplearning4j.org/, accessed on 4 November 2024) was used to train the models. As shown in Figure 2, four layers were set up. The input length of the first LSTM layer was equal to the encoding length of the AADs. The first layer’s activation function was “*tanh*”, the dropout value was 0.4, and the output length was 128. The input and output lengths of LSTM layers 2–3 were 128, the activation functions were all “*tanh*”, and the dropout values were 0.4 and 0.25, respectively. The input and output lengths of the output layer were 128 and 2, respectively, and the activation function was “*sigmoid*”.

### 2.4. Evaluation of Performance

Five evaluation indices were used to evaluate the prediction performance of the proposed models, including sensitivity (Sn), specificity (Sp), accuracy (Acc), Matthews’s correlation coefficient (MCC), and the area under the receiver operating characteristic curves (AUC). These indices were defined as follows:

(1) $$Sn=\frac{TP}{TP+FN}$$

(2) $$Sp=\frac{TN}{TN+FP}$$

(3) $$Acc=\frac{TP+TN}{TP+TN+FP+FN}$$

(4) $$MCC=\frac{TP\times TN-FP\times FN}{\sqrt{\left(TP+FP)(TP+FN)(TN+FP)(TN+FN\right)}}$$

Additionally, we used 0 to mask the single or multiple target parameters for all samples, then evaluated the importance score of features using the following formula:

(5) $$\mathrm{Importance}\;\mathrm{score}=\frac{FP+FN}{TP+TN+FP+FN}$$

where TP, FN, FP, and TN denote the numbers of correctly predicted ACEiPs, misclassified non-ACEiPs, misclassified ACEiPs, and correctly predicted non-ACEiPs, respectively.

### 2.5. Webserver Construction

ACEiPP was deployed on the Tencent Cloud Server (Windows Server 2012 R2 Standard Edition 64-bit) through Java web technology. The web directory was designed using HTML, JSP, and JavaScript. MySQL (version 5.7.16) was used for data storage and invoked by manually written Java programs to analyze the data. EChart (https://echarts.apache.org/en/index.html, accessed on 4 November 2024) technology was used for data visualization. ACEiPP can provide multiple services such as prediction, screening, and the structure–activity exploration of ACEiPs.

### 2.6. Theoretical Screening of Food-Derived ACEiPs

For this study, we used two different peptide libraries to screen potential ACEiPs.

A total of 4506 non-repetitive sequences of 31 kinds of food-derived bioactive peptides were derived from the DFBP [12], and a total of 2545 bioactive peptides with unknown ACE-inhibitory activity (e.g., antioxidant, DPP IV-inhibitory, antithrombotic, and anticancer activities) were obtained by excluding 1961 reported ACEiPs. ACEiPP was used to identify potential ACEiPs from these 2545 peptides.

The sequences of 21,249 food-derived proteins were retrieved from the DFBP, and the THP-Tool (http://www.cqudfbp.net/enzymes/hydrolysis_tools/dataInput.jsp, accessed on 4 November 2024) was used for the virtual hydrolysis of these proteins [12]. It is important to note that the virtual hydrolysis process assumes that hydrolysis occurs under optimal conditions, including ideal pH and temperature, to reflect the maximum enzyme activity. Six typical protein hydrolases, including chymotrypsin (EC 3.4.21.1), papain (EC 3.4.22.2), proteinase K (EC 3.4.21.64), thermolysin (EC 3.4.24.27), pepsin (EC 3.4.23.1), and trypsin (EC 3.4.21.4), were used to generate a large number of peptides, and the resulting peptides were predicted by ACEiPP to obtain potential food-derived ACEiPs.

### 2.7. Molecular Docking

The 3D crystal structure of human ACE, complexed with Lisinopril (PDB ID: 1O86.pdb), was retrieved from the Protein Data Bank (http://www1.rcsb.org/, accessed on 4 November 2024). The 3D structures of the peptides were manually constructed using Discovery Studio 2019 (Dassault Systèmes, BIOVIA, San Diego, CA, USA) and optimized for energy minimization using the “CHARMm 46b1” force field. The ACE’s active pocket was generated based on the binding site of the original ligand Lisinopril, and the semi-flexible docking of the ACE (rigid) and peptide (flexible) was performed using the “CDOCKER” module. The number of interaction poses that were generated per complex was set to 20, and the other parameters were used as default values. The first, top docking result of “-C DOCKER interaction energy” was chosen as the optimal binding of the peptide and ACE.

### 2.8. Determination of ACE Inhibitory Activity

The ACE inhibitory activity assay was performed according to the method reported by Lahogue and Cushman [30,31], with appropriate modifications. First, 10 μL of substrate (HHL, 5 mM, Sigma-Aldrich Co., Shanghai, China) was mixed with 100 μL of sample or sodium borate buffer (pH = 8.3, 0.1 M boric acid, 0.3 M NaCl, Sigma-Aldrich Co., Shanghai, China), and the reaction was initiated by the addition of 20 μL of ACE solution (0.1 U/L, Sigma-Aldrich Co., Shanghai, China) in a warm bath at 37 °C for 5 min. The samples, substrate HHL, and ACE solutions were prepared from sodium borate buffer. The peak areas of the mixtures were measured at 228 nm using an HPLC system (LC-20A, Shimadzu Co., Kyoto, Japan) and an InertSustain AQ-C18 column (4.6 × 250 mm, 5 μm particle size). The conditions of the HPLC analysis were as follows: mobile phase A (ultrapure water with 0.05% TFA, Merck & Co., Inc., Darmstadt, Germany) and mobile phase B (ACN, Merck & Co., Inc., Darmstadt, Germany); the volume ratio was 25:75, with isocratic elution at a flow rate of 1 mL/min for 11 min; the column temperature was 30 °C, and the injection volume was 10 μL. The ACE inhibition (%) was calculated according to the following formula:

(6) $$\mathrm{ACE}\;\mathrm{inhibitory}\;\mathrm{rate}\left(\%\right)=\frac{A_{blank}-A_{sample}}{A_{blank}}\times 100\%$$

where A_blank_ and A_sample_ represent the peak areas of the hippuric acid chromatographic peaks in the blank (sodium borate buffer) and in the sample, respectively. IC_50_ refers to the concentration value of the corresponding peptide sample (μM) when a 50% inhibition of ACE was achieved, and the value was calculated based on the regression curves of each component.

## 3. Results and Discussion

### 3.1. Performance of Single Coding Models

Twenty-two single AADs were used to convert the peptide sequences into matrices containing the sequence/structure characteristics as the input parameters of neural networks to train the LSTM models. As shown in the five-fold cross-validation conducted on the benchmark dataset (Supplementary Table S3), 18 models exhibited higher predictive performance, with Acc values of 0.7705–0.9308, MCC values of 0.5458–0.8629, and AUC values of 0.8653–0.9773, compared with the remaining four models (ISA-ECI, Lin’s scales, MS-WHIM, and VSTV), which were trained with three or two features. Moreover, 16 models (number of features: 6–20) exhibited high Acc values (0.8348–0.9211) on the test set (independent_ACEiPs), which contained 313 ACEiPs and 313 non-ACEiPs (Supplementary Table S4).

Thus, the LSTM-based models could effectively distinguish the dependencies between residue sites in unknown peptide sequences and accurately identify the key features of ACEiPs and non-ACEiPs. The models that learned 10, or more than 10, features generally showed higher predictive performance than the models based on fewer than 10 features. Each of the AADs translated different types and physicochemical attribute characteristics of residues encoded in peptide sequences, so multi-feature inputs could prompt LSTM models to reasonably learn the sequence/structure characteristics of peptides. Therefore, it is feasible to use single ADDs to extract the features in order to characterize peptide sequences and feed them into LSTM models. However, we must face a new challenge: optimizing and screening new sequence/structure characteristics with high efficiency but low redundancy.

### 3.2. Performance of Optimized Coding Models

**Figure 3:**
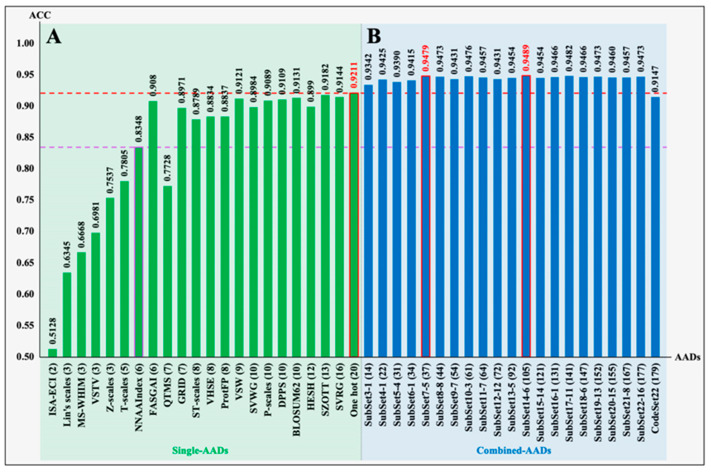
The predicted Acc on the independent dataset (independent_ACEiPs), based on different AADs: (**A**) 22 single AADs (Supplementary Table S4); (**B**) 22 optimized AADs (Supplementary Table S5). CodeSet22 is a total feature matrix consisting of 22 single AADs. Each “SubSet” represents the selected combined AADs gradually screened out from CodeSet22 by the “Leave-Group-Out” method. For example, SubSet22-16 represents the optimized encodings obtained by removing the 16th single AAD from CodeSet22 (see Supplementary Table S6 for the detailed screening process). The numbers in parentheses represent the number of features.

To further optimize a set of high-efficiency AADs with low redundancy, we restructured all single AADs into combined AADs as inputs of the LSTM model and evaluated the predictive performance of the trained models. First, the 22 single AADs were integrated into CodeSet22 to evaluate the Acc value of the obtained model. Then, “Leave-Group-Out” was used to reduce feature redundancy by gradually removing one group of single AADs from CodeSet22 in turn, and the optimal feature matrices for each round of training would be saved to perform the next optimization. The five-fold cross-validation scores (Supplementary Table S5) and independent test scores (Supplementary Table S6) indicated that the restructured combined AADs have more stable feature characterization ability than single AADs. As shown in Figure 3, the models based on combined AADs have the following key characteristics relative to the models based on single AADs. (i) Except for the model based on CodeSet22, the optimal models trained with combined AADs generated higher Acc values (from 0.9342 to 0.9489) than the model based on single AADs, even SubSet3-1 (combined with FASGAI and the ST scale; see Supplementary Table S6) with only 14 features generated higher Acc than all single AADs. (ii) With the increasing number of features from 2 to 37, the Acc values gradually improved, indicating that the effective features were continuously accumulated. (iii) When the number of features was added up to 37, SubSet7-5 (named VVSFZL37, composed of the FASGAI, Lin’s scales, ST scales, VHSE, VSW, and Z-scales) achieved a critical point whereby a set of non-redundant features were extracted from the total feature set (CodeSet22 with 179 features). The obtained model exhibited an Acc value of 0.9479, with an enhancement of 2.68% compared to the One-hot model with the highest Acc value in all the models by single AADs. (iv) With the increasing number of features (more than 37), the Acc values basically stabilized above 0.9431, revealing that the redundancy of features began to gradually increase. This not only made the variable weights increase ineffectively but also added to the difficulty of training; even the Acc values rapidly dropped to 0.9147 when the number of features accumulated to 179. Therefore, VVSFZL37 was the most efficient coding set for translating peptide sequences into high-efficiency features, which were used to train the LSTM model to acquire the predictor of ACEiPs.

### 3.3. Comparison with the Existing Predictors

To evaluate the generalization ability of ACEiPP with the existing predictors (i.e., mAHTPred, AHTpin_AAC, and AHTpin_ATC), we carried out comprehensive comparisons on three independent datasets. The evaluation indices on the independent_ACEiPs test set (Table 1) indicate that ACEiPP (Acc = 0.9479, MCC = 0.8959) achieved higher prediction performance than the three existing models. To be specific, ACEiPP showed the best prediction ability with an Acc value of 0.9479, while the Acc values of mAHTPred, AHTpin_AAC, and AHTpin_ATC were only 0.8492, 0.7706 (average), and 0.756 (average), respectively.

We then evaluated the predictive power of each model for AHTPs on the independent_AHTPs test set (Table 1). The Acc values of ACEiPP, mAHTPred, AHTpin_AAC, and AHTpin_ATC were 0.8303, 0.8834, 0.8061 (average), and 0.8204 (average), respectively, showing that ACEiPP has better prediction ability than AHTpin_ACC and AHTpin_ATC, but has poorer prediction ability than mAHTPred for AHTPs. As we know, AHTPs involve multi-type target protein inhibitors [32,33], which can be seen in the dataset independent_AHTPs, including ACEiPs and DPP IV-inhibitory, antioxidant, and antihypertensive peptides [22]. Nevertheless, ACEiPP was trained on the ACEiPs and non-ACEiPs benchmark dataset, meaning that ACEiPP only learned the features of ACEiPs but was unable to effectively identify the features of the whole AHTP samples, thereby affecting the prediction performance for AHTPs.

### 3.4. Key Sequence/Structure Characteristics Learned by ACEiPP

**Figure 4:**
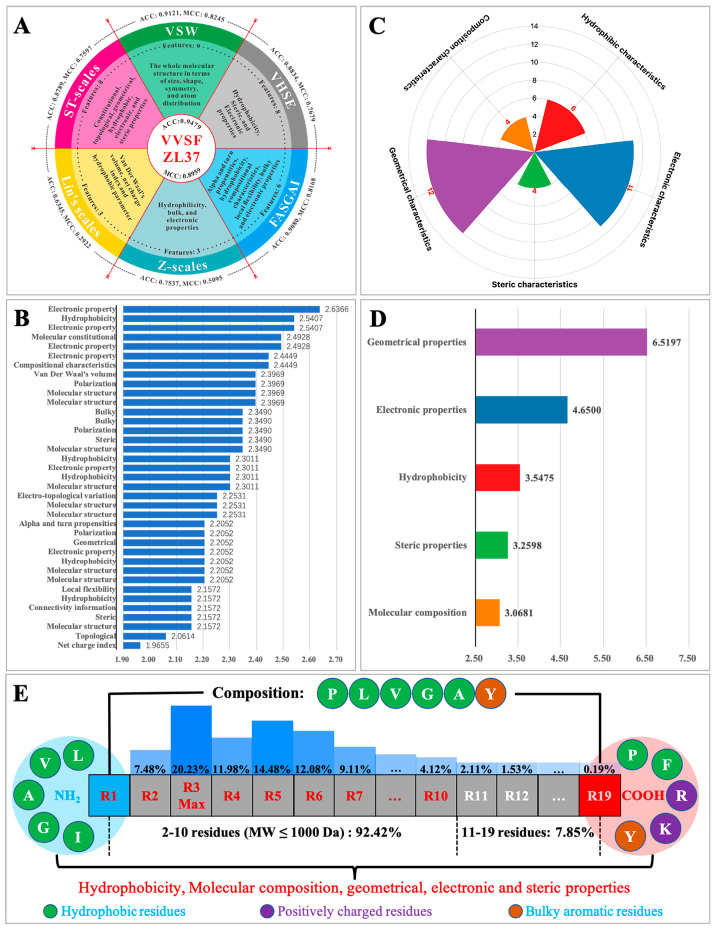
Feature composition and importance scores of VVSFZL37. (**A**) Six kinds of single AADs are contained in VVSFZL37, along with their physicochemical properties; (**B**) importance scores for 37 single features; (**C**) five patterns, determined by the physicochemical meaning; (**D**) importance scores for five patterns of features; (**E**) sequence characteristics of ACEiPs. ACEiPs are mainly oligopeptides (2–10 residues, molecular weight ≤ 1000 Da), composed of hydrophobic amino acids (Pro, Leu, Val, Gly, and Ala) and aromatic amino acids (Tyr); the C-terminus tends to be hydrophobic (Pro and Phe), positively charged (Arg and Lys), and bulky aromatic (Tyr) amino acids, while the N-terminus tends to be hydrophobic amino acids (Leu, Val, Ala, Gly, and Ile).

To explore the key characteristics learned by ACEiPP, we interpreted the physicochemical meaning of VVSFZL37 (Supplementary Table S7). As shown in Figure 4A, VVSFZL37 consists of 37 parameters derived from six sets of AADs (VSW, VHSE, ST-scales, Z-scales, FASGAI, and Lin’s scales), showing higher predictive power than any single AADs. All 37 parameters made important contributions to model performance, especially the top ten parameters, involving electronic properties, hydrophobicity, molecular composition and structure, and van der Waal volume, which were important residue characteristics of ACEiPs (Figure 4B). To further compare the overall contribution of the same/similar parameters to the model, we divided these 37 parameters into five patterns according to their physicochemical meanings, namely, geometrical, electronic, hydrophobic, steric, and composition characteristics, consisting of 12, 11, 6, 4, and 4 parameters, respectively (Figure 4C, Supplementary Table S8). Their importance scores are ordered as follows: Geometrical > Electronic > Hydrophobic > Steric > Composition (Figure 4D).

The five patterns of physicochemical attributes encoded by VVSFZL37 can also be intuitively observed in the sequence features of ACEiPs (Figure 4E). Several reports have indicated that hydrophobic, aromatic/aliphatic, C- and N-terminal, residue composition, and charged amino acids are indeed the key factors affecting the activities of ACEiPs. They not only directly determine the molecular geometry, overall hydrophobicity, side-chain functional groups, and charged characteristics of ACEiPs but also indirectly affect the second structure, steric structure, and molecular compositional characteristics of ACEiPs [2,3,17] (see Supplementary Note S1 for details). These studies strongly supported our conclusion that these five patterns of physicochemical attributes are indeed important factors in forming ACEiPs. Therefore, VVSFZL37 constructed plausible multiple properties and a low redundancy matrix to characterize various sequence/structure characteristics that are closely related to the activity of ACEiPs, thereby improving the robustness and accuracy of the LSTM model, rather than all single AADs.

### 3.5. Web Server Implementation

We built the ACEiPP server (http://www.cqudfbp.net/ACEiPP/index.jsp, accessed on 4 November 2024) with the optimal LSTM model, trained by VVSFZL37 to predict, design, and screen ACEiPs. ACEiPP comprises six modules, including Home, Pre-ACEiPs, Seq-Features, Pre-Libraries, Help, and Contact. As the first DL-based prediction platform for ACEiPs, ACEiPP can perform multiple applications such as prediction, residue-based mutation screening, structure–activity explorations of new ACEiPs, and the discovery of potential ACEiPs.

### 3.6. Characterization and Screening of Food-Derived ACEiPs with Multi-Activities

By selecting food-derived dipeptides or tripeptides in the DFBP that have been reported to have specific physiological functions but have not been reported to have anti-ACE inhibitory effects, we used ACEiPP prediction, combined with bioinformatics and molecular docking screening, to obtain bioactive peptides with anti-ACE effects. We used ACEiPP to predict the ACE inhibitory activity of 2346 peptides and obtained 1358 potential ACEiPs (probability threshold > 0.5). Based on Lipinski’s rule of five, combined with TPSA, water solubility, GI, P-gp substrate, BBBP, CYP enzyme inhibition, and toxicity predictions, we obtained 37 food-derived dipeptides and tripeptides (Supplementary Table S9).

The docking results of 37 peptides with the target protein ACE showed that 19 of the peptide-ACE scores were higher than Lisinopril (83.7945 kcal/mol) (Supplementary Table S10), implying that these peptides may have a strong inhibitory effect on ACE (the type and number of non-bonding forces of the eight highest-scoring peptides with ACE are shown in Supplementary Table S11). These eight peptides have a similar binding mode to Lisinopril (Supplementary Figure S1), i.e., they bind primarily through hydrogen bonds (including a conventional hydrogen bond, salt bridge, carbon–hydrogen bond, and Pi-donor hydrogen bond) and non-hydrogen bonds (Pi-Cation, attractive charge, and Pi-Alkyl bonds) to promote inhibitory peptide interactions with S1 (Ala354, Glu384, and Tyr523), S2 (His353 and His513), S1’ (Glu162), and the Zn^2+^-binding domain (His383) of the ACE. In addition, these eight peptides can also form the same attractive charge or metal acceptor with metal ions (Zn701) as Lisinopril, thus hindering the activating effect of Zn701 on ACE.

The experimental effects of eight peptides on ACE inhibition (Table 2) shows that four peptides, namely, LAF, LLL, GLF, and LIV, exhibit relatively high ACE inhibitory activities, with IC_50_ values of 4.35 μM, 17.99 μM, 270.93 μM, and 330.75 μM, respectively. By reviewing the DFBP, we found that these four peptides also possess 1 (antioxidative), 3 (antioxidative, DPP IV-inhibitory, and stimulating), 3 (antimicrobial, immunomodulatory, and regulating) and 1 (antioxidative) reported biological activities, respectively. The other four peptides (LAL, AVL, LE, and VLV) show low/no ACE inhibitory activity (IC_50_ > 1000 μM).

### 3.7. Future Development Trends for ACEiPs

We used ACEiPP to predict 1,320,635 potential ACEiPs that are encoded in food-derived proteins (http://www.cqudfbp.net/ACEiPP/prePool/dataPool.jsp, accessed on 4 November 2024), and further compared the sequence features between the experimental ACEiPs (IC_50_ < 1000 μM; 1043 unique sequences) and the predicted potential ACEiPs. The experimentally discovered ACEiPs are concentrated in the tripeptides to octapeptides (mainly tripeptides and pentapeptides), whereas the predicted ACEiPs were primarily concentrated in the pentapeptides to octapeptides, with the most being discovered for hexapeptides (Supplementary Figure S2). This difference may be attributed to the limitations of the actual enzymatic hydrolysis conditions and the number of reports. Prospectively, the main distribution area of ACEiPs may be concentrated in the range of pentapeptides to octapeptides, especially hexapeptides, providing an important reference for the screening of ACEiPs in the future. This is because small peptides (2–4 residues) can access the active pocket of ACE but cannot form enough hydrogen bonds, while large peptides (≥9 residues) cannot enter the active pocket easily, and it may be hard to change the catalytic activity of ACE. Medium peptides (5–8 residues) might be the most suitable, which is partly due to the contribution of hydrogen bonds to their affinity [34,35]. Furthermore, the predicted ACEiPs have similar residue composition and two-terminal features to experimental ACEiPs (Supplementary Figure S2). Therefore, we not only demonstrated the feature consistency between predicted and experimental ACEiPs but also answered two open questions in ACEiP research: (i) the relationship between peptide chain length and ACE-inhibitory activity; (ii) the optimal amino acid profiles of ACEiPs that are derived from food proteins [20].

## 4. Conclusions

For this study, we developed a predictor ACEiPP using a DL-based model based on natural sequences processing by LSTMs and used the interpretability of AADs to predict, design, and screen ACEiPs. ACEiPP effectively learned the key sequence/structure characteristics of ACEiPs for transformation by VVSFZL37 and shows improved prediction performance compared with the existing three models. The optimal sequence/configuration profiles affecting the ACE inhibitory activity of the peptides were mapped. We used ACEiPP to acquire four ACEiPs, namely, LAF, LLL, GLF, and LIV, with other known bioactivities (hypertension, diabetes, hyperlipidemia, etc.), indicating that ACEiPP can facilitate the efficient discovery of bioactive peptides. In the future, our method may be applied to other DL-based studies on peptides for transfer learning.

## Figures and Tables

**Table 1:** Performance comparison of ACEiPP and the three existing predictors on independent_ACEiPs and independent_AHTPs.

Predictor ^a^	Applicable Length	Independent_ACEiPs		Independent_AHTPs	
		Acc	MCC	Acc	MCC
**AHTpin_AAC**	Tetrapeptides	0.7639	0.5168	-	-
	Pentapeptides	0.5429	0.0880	0.7814	0.4121
	Hexapeptides	0.8507	0.7152	0.9045	0.4354
	Medium peptides (7–12)	0.7957	0.5898	0.7622	0.5033
	Large peptides (≥13)	0.9000	0.7980	0.7761	0.2618
AHTpin_ATC	Tetrapeptides	0.7639	0.5168	-	-
	Pentapeptides	0.5810	0.1674	0.7705	0.3292
	Hexapeptides	0.7910	0.5775	0.9045	0.3582
	Medium peptides (7–12)	0.8441	0.7053	0.7744	0.4828
	Large peptides (≥13)	0.8000	0.6162	0.8321	0.3256
mAHTPred	≥5	0.8492	0.7027	0.8834	0.7670
ACEiPP	≥2	0.9479	0.8959	0.8303	0.6614

^a^ AHTpin_AAC and AHTpin_ATC are two AHTP models that were trained with amino acid composition and atomic composition features, respectively. Since their evaluation parameters are determined according to different peptide lengths, the average values were calculated to compare their prediction performance. mAHTPred is an AHTP prediction model based on an extremely randomized tree, which can only predict sequences greater than or equal to five residues.

**Table 2:** The ACE inhibitory activity and multi-bioactivities of eight screened peptides.

No.	Sequence	IC_50_ (μM)	Bioactivities
1	LAF	4.35	High ACE-inhibitory activity Antioxidant activity (DFBPANOX1165)
2	LLL	17.99	High ACE-inhibitory activity Antioxidant activity (DFBPANOX0810) DPP IV-inhibitory activity (DFBPDPIV0160) Stimulating activity (DFBPSTPE0004) Multifunctional activity (DFBPMUFU0682)
3	GLF	270.93	Moderate ACE-inhibitory activity Antimicrobial activity (DFBPAMIC0518) Immunomodulatory activity (DFBPIMMU0002) Immunomodulatory activity (DFBPIMMU0093) Regulating activity (DFBPREPE0005) Multifunctional activity (DFBPMUFU0716)
4	LIV	330.75	Moderate ACE-inhibitory activity Antioxidant activity (DFBPANOX0821)
5	LAL	1162.34	Low ACE-inhibitory activity Antioxidant activity (DFBPANOX0812)
6	AVL	5093.25	No ACE-inhibitory activity Antihypertensive activity (DFBPANHY0635)
7	LE	28,526.40	No ACE-inhibitory activity Antiviral activity (DFBPANPE0012)
8	VLV	No inhibition	No ACE-inhibitory activity Antioxidant activity (DFBPANOX0910)

## Data Availability

The original contributions presented in the study are included in the article/Supplementary Materials; further inquiries can be directed to the corresponding author.

## Supplementary Materials

The following supporting information can be downloaded at: https://www.mdpi.com/article/10.3390/foods13223550/s1 [17,18,19,20,36,37,38,39,40,41,42,43,44,45,46,47,48,49,50,51,52,53,54,55,56,57,58], Figure S1: 2D-diagram of the interaction between eight pre-MBPs and ACE; Figure S2: Hydrolyzed peptide prediction (1,320,635 unique sequences, probability ≥ 0.5) and their sequence feature comparison with experimental ACEiPs (1043 unique sequences); Table S1: Benchmark and independent datasets; Table S2: The list of 22 AAD encodings and their parameter meanings collected from literature; Table S3: Five-fold cross-validation metrics comparison for 22 single-AADs on the benchmark dataset (benchmark_ACEiPs.txt); Table S4: The average performance evaluation scores for 22 single-AADs on the independent dataset (independent_ACEiPs.txt); Table S5: The average performance evaluation scores for combined-AADs on the benchmark dataset (benchmark_ACEiPs.txt); Table S6: The average performance evaluation scores for combined-AADs on the independent dataset; Table S7: The main coding features of VVSFZL37 consisting of six single-AADs; Table S8: Five classifications with similar/same characteristics in VVSFZL37; Table S9: Potential peptides screened based on ADMET; Table S10: The semi-flexible docking results of ACE receptor (rigid) with the different ligands (flexible); Table S11: Interaction of ACE receptors with eight peptides.
