# Supplementary material for: Sequence–Activity Relationship of Angiotensin-Converting Enzyme Inhibitory Peptides Derived from Food Proteins, Based on a New Deep Learning Model
Source: Foods. 2024 Nov 7;13(22):3550. doi: 10.3390/foods13223550 (PMC11592644; doi:10.3390/foods13223550)
Supplement: Supplementary file 1 [file foods-13-03550-s001.zip › foods-3276114-supplementary.pdf]

Supplementary Material

Sequence-activity relationship of food-derived angiotensin-converting enzyme inhibitory peptides based on a new deep learning model

Dongya Qin[#], Xiao Liang[#], Linna Jiao, Ruihong Wang, Yi Zhao, Wenjun Xue, and Guizhao Liang[*]

Key Laboratory of Biorheological Science and Technology, Ministry of Education, College of Bioengineering, Chongqing University, Chongqing 400044, China.

Note S1. Sequence Characterization of ACEiPs Related to ACE Inhibitory Activity. Hydrophobic, aromatic/aliphatic, C- and N-terminal, residue composition, and charged amino acids are the key factors affecting the activities of ACEiPs. Wu, etc. found dipeptides, tripeptides, and tetrapeptides tended to amino acid residues with (i) bulky aromatic amino acids as well as hydrophobic side chains, (ii) aromatic amino acids-positively charged amino acids-hydrophobic amino acids at the C-terminus, middle and N-terminus, and (iii) the amino acid pattern: C1 (Tyr, Pro, and Phe), C2 (Phe), C3 (Arg, His, Trp, and Phe), C4 (Val, Ile, and Met), respectively (Wu, Aluko, & Nakai, 2006). Sagardia, etc. proposed favorable amino acids for each C-terminal pentapeptide: C1 (Gly, Leu, Ala, Val, and Ile), C2 (Arg, Val, and Thr), C3 (Asp, Asn, and Lys), C4 (Trp, Tyr, and Cys) and C5 (Val and Ile) and indicated that the position C1 was the most relevant position in the model (followed by position C4) (Sagardia, Roa-Ureta, & Bald,

2013). Hernández, etc. reviewed C-terminal tripeptide residues of longer peptides dominate in competitive binding to the ACE active sites (Hernández-Ledesma, del Mar Contreras, & Recio, 2011). Aluko, Hernández, Kapel, etc. reported the branched-chain aliphatic amino acids (Leu, Ile, and Val), aromatic amino acids (Trp and Phe) and Pro at the C-terminus contribute to the increase in the ACE inhibitory ability of peptides. Lu, etc. indicated that the presence of N-terminal aliphatic amino acids (Val, Leu, Gly, Ile, and Ala) is positively correlated with the ACE inhibitory activity of peptides. Especially, when the N-terminus of peptides contains repeating aliphatic amino acids, the ACE inhibitory activity is more significantly improved (Xiang, Qiu, Zhao, Zheng, & Qiao, 2021).

Table S1. Benchmark and independent datasets

Predictors	Datasets	Positive	Negative	Length	Activity	Download
ACEiPP	benchmark_ACEiPs.txt	730	730	2-19	ACE-inhibitory	http://www.cqudfbp.net/ACEiPP/seqFeatures/dataSets.jsp
	independent_ACEiPs.txt	313	313	2-19	ACE-inhibitory	
mAHTPred	independent_AHTPs.txt	386	386	5-81	antihypertensive	http://thegleelab.org/mAHTPred/mAHTPredData.html

Table S2. The list of 22 AAD encodings and their parameter meanings collected from literature

No.	Feature descriptor	Dimension	Explanation	Reference
1	One hot	20	One-hot encoding represents each amino acid with a 20×1 vector with a single position (corresponding to the specific amino acid) set to one, and all 19 other positions set to zero. Each peptide was therefore represented by a 2D array created by concatenating the 20 × 1 vectors of the amino acids.	(Jurtz, Johansen, Nielsen, Almagro Armenteros, Nielsen, Sønderby, et al., 2017)
2	DPPS	10	The DPPS descriptors for 20 amino acids are derived by PCA. The electronic properties of the amino acid are characterized by V_1-V_4, steric properties by V_5 and V_6, hydrophobic properties by V_7 and V_8, and hydrogen bond contributions by V_9 and V_{10}.	(F. Tian, Yang, Lv, Yang, & Zhou, 2009)
3	BLOSUM62	10	The BLOSUM matrix-derived descriptors (BLOSUM) including 10 indices, representing hydrophobicity, alpha-helix propensity, beta-sheet propensity, bulkiness, charge, and composition, are based on both physicochemical properties that have been subjected to a ARIMAX analyses and an alignment matrix of 20 coded amino acids, the BLOSUM62 matrix.	(Georgiev, 2009; Henikoff & Henikoff, 1992)

4	FASGAI	6	Through factor analysis, the FASGAI descriptors cluster 335 physicochemical properties of each of 20 coded amino acids into 6 factors, which are related to hydrophobicity, alpha and turn propensities, bulky properties, compositional characteristics, local flexibility and electronic properties, respectively.	(Guizhao Liang & Li, 2007)
5	GRID	7	The 7 principal properties of the GRID scales are derived from PCA on interaction energies of 20 coded amino acids, with six different probes mimicking various functional groups which can be involved in peptide-peptide interactions.	(Cocchi & Johansson, 1993)
6	HESH	12	These 12 vectors of HESH for 20 amino acids were obtained by PCA. The v_1–v_4 were hydrophobic properties, v_5–v_6 were steric properties, v_7–v_{10} were electronic properties, and v_{11}–v_{12} were hydrogen bond contribution properties.	(Mao Shu, Mei, Yang, Liao, & Li, 2009)
7	ISA-ECI	2	The isotropic surface area (ISA) approximates the hydrophobic character of the side chain substituent; [b] the electronic charge index (ECI) is a measure of the charge concentration of the amino acid. Each residue was described by a combination of ISA and ECI descriptors.	(Collantes & Dunn, 1995)
8	Lin’s scales	3	Three kinds of physicochemical parameters selected from properties of 20 coded amino acids, namely Van Der Waal’s volume, net charge index and hydrophobic parameter, construct the vectors to characterize the structures of peptides.	(Lin, Long, Bo, Wang, & Wu, 2008)

9	MS-WHIM	3	MS-WHIM indexes, which are three principal components derived from PCA, are a collection of 36 statistical indexes aimed at extracting and condensing steric and electrostatic 3D-properties of a molecule.	(Zaliani & Gancia, 1999)
10	NNAAIndex	6	The NNAAIndex scales characterize a total of 155 physiochemical properties of 22 natural and 593 non-natural amino acids, followed by clustering the structural matrix into 6 representative property patterns by factor analysis. The six factors are geometric characteristics, H-bond, connectivity, accessible surface area, integy moments index, and volume and shape, respectively.	(G. Liang, Liu, Shi, Zhao, & Zheng, 2013)
11	ProtFP	8	The 8 principal component scores based on a PCA analysis of 58 amino acids properties explain 92% of the variances. For these predominant components, the first one is related to hydrophobicity and the second one is related to the size of amino acids.	(van Westen, Swier, Wegner, Ijzerman, van Vlijmen, & Bender, 2013)
12	QTMS (ADFQ) indices	7	The amino acids indices of QTMS are obtained by application of PCA on the unfolded loadings of a data matrix of QTMS of all bonds of amino acids.	(Hemmateenejad, Yousefinejad, & Mehdipour, 2011)

13	ST-scales	8	ST-scales, the 8 principal component scores of ST-scales are derived from 827 structural variables of 167 amino acids by PCA, and these parameters are mainly related to constitutional, topological, geometrical, hydrophobic, electronic, and steric properties of the amino acids	(Yang, Shu, Ma, Mei, Jiang, & Li, 2010)
14	SVRG	16	The 16 principal component scores are derived from PCA of 150 radial distribution function and 74 geometrical descriptors.	(Tong, Che, Li, Wang, Xu, & Chen, 2011)
15	SVWG	10	The 10 principal component scores are obtained by PCA on 99 WHIM and 197 GETWAY descriptors.	(Tong, Chen, Liu, Che, & Xu, 2012)
16	SZOTT	13	The SZOTT descriptors are derived from PCA of a matrix of 1369 structural variables including 0D, 1D, 2D and 3D information for 20 coded amino acids.	(G.-Z. Liang, Shu, & Li, 2008)
17	T-scales	5	The 5 principal component scores of T-scales are derived from PCA on the collected 67 kinds of structural and topological variables of 135 amino acids.	(Feifei Tian, Zhou, & Li, 2007)

18	VHSE	8	The VHSE descriptors are derived from the PCA on independent families of 18 hydrophobic properties, 17 steric properties, and 15 electronic properties, respectively. Among them, $VHSE_1$ and $VHSE_2$ are related to hydrophobic properties, $VHSE_3$ and $VHSE_4$ to steric properties, and $VHSE_5$–$VHSE_8$ to electronic properties.	(Mei, Liao, Zhou, & Li, 2005)
19	VSTV	3	The 3 principal component scores as VSTV descriptors are derived from PCA on a matrix of 25 structural and topological variables of 20 coded amino acids.	(Tong, Li, Bai, & Li, 2017)
20	VSW	9	VSW was derived from the principal component analysis of a matrix of 99 weighted holistic invariant molecular indices of amino acids, and contained information about the whole molecular structure in terms of size, shape, symmetry, and atom distribution.	(Tong, Liu, Zhou, Wu, & Li, 2008)
21	Z-scales	3	The 3 principal component scores are derived from PCA of a matrix of 29 physicochemical variables for 20 coded amino acid are related to hydrophilicity (z_1), bulk (z_2), and electronic properties (z_3).	(Hellberg, Sjostrom, Skagerberg, & Wold, 1987)
22	P-scales	10	P-scale was recruited as a novel set of physicochemical descriptors derived from component analysis on four short of physicochemical properties variables (hydrophobic, electronic, steric and hydrogen bond contribution) of 20 coded amino acids.	(M. Shu, Cheng, Zhang, Wang,

				Lin, Wang, et al., 2011)

Table S3. Five-fold cross-validation metrics comparison for 22 single-AADs on the benchmark dataset (benchmark_ACEiPs.txt)

No.	Descriptor	Matrix	*ACC*	*MCC*	*SEN*	*SPE*	*PRE*	*F1 Score*	*AUC*
1	One hot	20×20	0.9137	0.8287	0.8959	0.9315	0.9301	0.9123	0.9685
2	SVRG	20×16	0.9308	0.8629	0.9329	0.9288	0.9308	0.9311	0.9773
3	SZOTT	20×13	0.9192	0.8386	0.9219	0.9164	0.9172	0.9194	0.9735
4	HESH	20×12	0.9041	0.8092	0.9233	0.8849	0.8897	0.9059	0.9683
5	SVWG	20×10	0.9110	0.8229	0.9288	0.8932	0.8976	0.9126	0.9712
6	DPPS	20×10	0.9103	0.8218	0.8918	0.9288	0.9271	0.9087	0.9689
7	P-scales	20×10	0.8973	0.7952	0.8781	0.9164	0.9131	0.8952	0.9627
8	BLOSUM62	20×10	0.8911	0.7837	0.9178	0.8644	0.8717	0.8939	0.9627
9	VSW	20×9	0.9144	0.8292	0.9068	0.9219	0.9213	0.9138	0.9728
10	ProtFP	20×8	0.8699	0.7411	0.8849	0.8548	0.8606	0.8720	0.9515
11	ST-scales	20×8	0.8692	0.7392	0.8767	0.8616	0.8641	0.8699	0.9492
12	VHSE	20×8	0.8610	0.7235	0.8836	0.8384	0.8461	0.8639	0.9453
13	GRID	20×7	0.8966	0.7934	0.9055	0.8877	0.8897	0.8975	0.9590
14	QTMS	20×7	0.8429	0.6862	0.8596	0.8262	0.8320	0.8455	0.9231
15	FASGAI	20×6	0.9034	0.8075	0.8890	0.9178	0.9161	0.9022	0.9655
16	NNAAIndex	20×6	0.8349	0.6747	0.7863	0.8836	0.8735	0.8264	0.9134
17	T-scales	20×5	0.7705	0.5458	0.7397	0.8014	0.7921	0.7618	0.8653
18	Z-scales	20×3	0.7760	0.5580	0.8466	0.7055	0.7420	0.7907	0.8417
19	VSTV	20×3	0.6877	0.3798	0.7493	0.6260	0.6634	0.7033	0.7487
20	MS-WHIM	20×3	0.6582	0.3246	0.7630	0.5534	0.6306	0.6901	0.7219
21	Lin's scales	20×3	0.6212	0.2677	0.8521	0.3904	0.5915	0.6952	0.6743
22	ISA-ECI	20×2	0.5699	0.1784	0.3184	0.8231	0.6810	0.3962	0.6659

Table S4. The average performance evaluation scores for 22 single-AADs on the independent dataset (independent_ACEiPs.txt)

No.	Descriptor	Matrix	*ACC*	*MCC*	*SEN*	*SPE*	*PRE*	*F1 Score*	*AUC*
1	One hot	20×20	0.9211	0.8429	0.9099	0.9323	0.9312	0.9202	0.9743
2	SVRG	20×16	0.9144	0.8294	0.9246	0.9042	0.9070	0.9155	0.9726
3	SZOTT	20×13	0.9182	0.8367	0.9310	0.9054	0.9079	0.9193	0.9764
4	HESH	20×12	0.8990	0.7995	0.9265	0.8716	0.8786	0.9018	0.9710
5	BLOSUM62	20×10	0.9131	0.8269	0.9310	0.8952	0.8991	0.9146	0.9745
6	DPPS	20×10	0.9109	0.8224	0.8933	0.9284	0.9260	0.9093	0.9713
7	P-scales	20×10	0.9089	0.8182	0.9067	0.9112	0.9111	0.9087	0.9715
8	SVWG	20×10	0.8984	0.7978	0.9208	0.8760	0.8816	0.9006	0.9714
9	VSW	20×9	0.9121	0.8245	0.9048	0.9195	0.9183	0.9114	0.9725
10	ProtFP	20×8	0.8837	0.7687	0.9105	0.8569	0.8642	0.8867	0.9600
11	VHSE	20×8	0.8834	0.7679	0.9080	0.8588	0.8656	0.8862	0.9538
12	ST-scales	20×8	0.8789	0.7597	0.8939	0.8639	0.8697	0.8808	0.9494
13	GRID	20×7	0.8971	0.7949	0.8990	0.8952	0.8966	0.8974	0.9598
14	QTMS	20×7	0.7728	0.5467	0.7617	0.7840	0.7798	0.7700	0.8446
15	FASGAI	20×6	0.9080	0.8168	0.8888	0.9272	0.9243	0.9061	0.9703
16	NNAAIndex	20×6	0.8348	0.6726	0.7955	0.8741	0.8643	0.8279	0.9106
17	T-scales	20×5	0.7805	0.5672	0.7553	0.8058	0.8010	0.7730	0.8680
18	Z-scales	20×3	0.7537	0.5095	0.7987	0.7086	0.7329	0.7643	0.8410
19	VSTV	20×3	0.6981	0.3999	0.7444	0.6518	0.6779	0.7086	0.7592
20	MS-WHIM	20×3	0.6668	0.3428	0.7738	0.5597	0.6380	0.6986	0.7376
21	Lin's scales	20×3	0.6345	0.2922	0.8530	0.4160	0.6033	0.7036	0.6901
22	ISA-ECI	20×2	0.5128	0.0308	0.2332	0.7923	0.5290	0.3237	0.5792

Table S5. The average performance evaluation scores for combined-AADs on the benchmark dataset (benchmark_ACEiPs.txt)

Opt. [a]	Removed AADs [b]	Opt_AADs [c]	Matrix	*ACC*	*MCC*	*SEN*	*SPE*	*PRE*	*F1 Score*	*AUC*
1	No deletion (0)	SubSet22	20×179	0.9178	0.8364	0.9041	0.9315	0.9298	0.9165	0.9738
2	ISA-ECI (2)	SubSet22-16	20×177	0.9377	0.8756	0.9288	0.9466	0.9458	0.9371	0.9851
3	SVWG (10)	SubSet21-8	20×167	0.9438	0.8879	0.9397	0.9479	0.9476	0.9435	0.9875
4	HESH (12)	SubSet20-15	20×155	0.9397	0.8796	0.9425	0.937	0.9376	0.9400	0.9873
5	MS-WHIM (3)	SubSet19-13	20×152	0.9363	0.8728	0.9356	0.937	0.9372	0.9363	0.9854
6	T-scales (5)	SubSet18-6	20×147	0.9432	0.8868	0.9301	0.9562	0.9551	0.9424	0.9848
7	NNAAIndex (6)	SubSet17-11	20×141	0.9541	0.9090	0.9411	0.9671	0.9667	0.9535	0.9898
8	P-scales (10)	SubSet16-1	20×131	0.9349	0.8707	0.9315	0.9384	0.9391	0.9348	0.9838
9	BLOSUM62 (10)	SubSet15-14	20×121	0.9356	0.8718	0.9315	0.9397	0.9393	0.9351	0.9857
10	SVRG (16)	SubSet14-6	20×105	0.9445	0.8891	0.9425	0.9466	0.9464	0.9444	0.9883
11	SZOTT (13)	SubSet13-5	20×92	0.9438	0.8882	0.9425	0.9452	0.9458	0.9439	0.9874
12	One hot (20)	SubSet12-12	20×72	0.9514	0.9032	0.9425	0.9603	0.9600	0.9510	0.9880
13	ProtFP (8)	SubSet11-7	20×64	0.9445	0.8893	0.9384	0.9507	0.9502	0.9441	0.9867
14	VSTV (3)	SubSet10-3	20×61	0.9438	0.8883	0.9384	0.9493	0.9494	0.9435	0.9874
15	GRID (7)	SubSet9-7	20×54	0.9438	0.8878	0.9452	0.9425	0.9426	0.9439	0.9858
16	DPPS (10)	SubSet8-8	20×44	0.9459	0.8924	0.9370	0.9548	0.9545	0.9454	0.9851

17	QTMS (7)	SubSet7-5 (=VVSFZL37)	20×37	0.9507	0.9017	0.9548	0.9466	0.9473	0.9509	0.9885
18	Z-scales (3)	SubSet6-1	20×34	0.9397	0.8797	0.9466	0.9329	0.934	0.9402	0.9843
19	Lin's scales (3)	SubSet5-4	20×31	0.9349	0.8700	0.9356	0.9342	0.9346	0.9350	0.9821
20	VSW (9)	SubSet4-1	20×22	0.9418	0.8846	0.9479	0.9356	0.9377	0.9423	0.9863
21	VHSE (8)	SubSet3-1	20×14	0.9390	0.8791	0.9329	0.9452	0.9456	0.9387	0.9819
22	ST-scales (8)	SubSet2-1 (=FASGAI)	20×6	0.9034	0.8075	0.8890	0.9178	0.9161	0.9022	0.9655

[a] Opt.: Number of optimizations; [b] Delete AADs: Redundant encodings that are deleted during each round of optimization by using "Leave-Group-Out", and the number in each parentheses indicates the encoded length of the removed AAD; [c] Opt_AADs: The optimal feature encodings screened out by "Leave-Group-Out" in each round of training.

Table S6. The average performance evaluation scores for combined-AADs on the independent dataset (independent_ACEiPs.txt)

Opt. [a]	Removed AADs [b]	Opt_AADs [c]	Matrix	*ACC*	*MCC*	*SEN*	*SPE*	*PRE*	*F1 Score*	*AUC*
1	No deletion (0)	SubSet22	20×179	0.9198	0.8398	0.9265	0.9131	0.9143	0.9203	0.9752
2	ISA-ECI (2)	SubSet22-16	20×177	0.9473	0.8947	0.9425	0.9521	0.9517	0.9470	0.9853
3	SVWG (10)	SubSet21-8	20×167	0.9457	0.8915	0.9489	0.9425	0.9429	0.9458	0.9868
4	HESH (12)	SubSet20-15	20×155	0.9460	0.8921	0.9495	0.9425	0.9430	0.9462	0.9862
5	MS-WHIM (3)	SubSet19-13	20×152	0.9473	0.8946	0.9470	0.9476	0.9476	0.9473	0.9863
6	T-scales (5)	SubSet18-6	20×147	0.9466	0.8934	0.9476	0.9457	0.9458	0.9467	0.9866
7	NNAAIndex (6)	SubSet17-11	20×141	0.9482	0.8966	0.9482	0.9482	0.9484	0.9483	0.9876
8	P-scales (10)	SubSet16-1	20×131	0.9466	0.8934	0.9450	0.9482	0.9482	0.9466	0.9870
9	BLOSUM62 (10)	SubSet15-14	20×121	0.9454	0.8908	0.9463	0.9444	0.9446	0.9454	0.9861
10	SVRG (16)	SubSet14-6	20×105	0.9489	0.8979	0.9457	0.9521	0.9520	0.9487	0.9877
11	SZOTT (13)	SubSet13-5	20×92	0.9454	0.8909	0.9482	0.9425	0.9430	0.9455	0.9877
12	One hot (20)	SubSet12-12	20×72	0.9431	0.8863	0.9406	0.9457	0.9455	0.9430	0.9872
13	ProtFP (8)	SubSet11-7	20×64	0.9457	0.8915	0.9463	0.9450	0.9452	0.9457	0.9853
14	VSTV (3)	SubSet10-3	20×61	0.9476	0.8953	0.9450	0.9502	0.9500	0.9475	0.9865
15	GRID (7)	SubSet9-7	20×54	0.9431	0.8863	0.9431	0.9431	0.9432	0.9432	0.9858
16	DPPS (10)	SubSet8-8	20×44	0.9473	0.8946	0.9463	0.9482	0.9482	0.9472	0.9857

17	QTMS (7)	SubSet7-5 (=FLSVVZ37)	20×37	0.9479	0.8959	0.9495	0.9463	0.9466	0.9480	0.9876
18	Z-scales (3)	SubSet6-1	20×34	0.9415	0.8832	0.9457	0.9374	0.9380	0.9418	0.9840
19	Lin's scales (3)	SubSet5-4	20×31	0.9390	0.8781	0.9444	0.9335	0.9343	0.9393	0.9829
20	VSW (9)	SubSet4-1	20×22	0.9425	0.8854	0.9489	0.9361	0.9374	0.9429	0.9856
21	VHSE (8)	SubSet3-1	20×14	0.9342	0.8684	0.9335	0.9348	0.9347	0.9341	0.9824
22	ST-scales (8)	SubSet2-1 (=FASGAI)	20×6	0.9080	0.8168	0.8888	0.9272	0.9243	0.9061	0.9703

[a] Opt.: Number of optimizations; [b] Delete AADs: Redundant encodings that are deleted during each round of optimization by using "Leave-Group-Out", and the number in each parentheses indicates the encoded length of the removed AAD; [c] Opt_AADs: The optimal feature encodings screened out by "Leave-Group-Out" in each round of training.

Table S7. The main coding features of VVSFZL37 consisting of six single-AADs

Variables	Sequence/structure characteristics	AADs[a]	Method	Matrix	*ACC*
Var_1					
Var_2					
Var_3					
Var_4	The whole molecular structure in terms of size, shape, symmetry, and atom distribution	VSW	PCA[b]	20×9	0.9121
Var_5					
Var_6					
Var_7					
Var_8					
Var_9					
Var_10	Hydrophobicity	VHSE	PCA	20×8	0.8834
Var_11	Hydrophobicity				
Var_12	Steric properties				
Var_13	Steric properties				
Var_14	Electronic property				
Var_15	Electronic property				
Var_16	Electronic property				
Var_17	Electronic property				
Var_18	Molecular constitutional, topological, geometrical, connectivity information, atomic molecular electro-topological variation, and polarization	ST-scales	PCA	20×8	0.8789
Var_19					
Var_20					
Var_21					
Var_22					
Var_23					
Var_24					
Var_25					

Var_26	Hydrophobicity	FASGAI	FA[c]	20×6	0.9080
Var_27	Alpha and turn propensities				
Var_28	Bulky properties				
Var_29	Compositional characteristics				
Var_30	Local flexibility				
Var_31	Electronic property				
Var_32	Hydrophilicity	Z-scales	PCA	20×3	0.7537
Var_33	Bulky properties				
Var_34	Electronic property				
Var_35	Van Der Waal's volume	Lin's scales	MC[d]	20×3	0.6345
Var_36	Net charge index				
Var_37	Hydrophobicity				

[a] AADs: Amino acid descriptors; [b] PCA: principal components analysis; [c] FA: factor analysis. [d] MC: Manual collection.

Table S8. Five classifications with similar/same characteristics in VVSFZL37

Classification	Variables	AADs[a]
Geometrical	Var_1	VSW
	Var_2	VSW
	Var_3	VSW
	Var_4	VSW
	Var_5	VSW
	Var_6	VSW
	Var_7	VSW
	Var_8	VSW
	Var_9	VSW
	Var_27	FASGAI
	Var_30	FASGAI
Electronic	Var_14	VHSE
	Var_15	VHSE
	Var_16	VHSE
	Var_17	VHSE
	Var_22	ST-scales
	Var_23	ST-scales
	Var_24	ST-scales
	Var_25	ST-scales
	Var_31	FASGAI
	Var_34	Z-scales
	Var_36	Lin's scales
Hydrophobic	Var_10	VHSE
	Var_11	VHSE
	Var_26	FASGAI

	Var_32	Z-scales
	Var_35	Lin's scales
	Var_37	Lin's scales
Steric	Var_12	VHSE
	Var_13	VHSE
	Var_28	FASGAI
	Var_33	Z-scales
Composition	Var_18	ST-scales
	Var_19	ST-scales
	Var_21	ST-scales
	Var_29	FASGAI

[a] AADs: Amino acid descriptors

Table S9. Potential peptides screened based on ADMET

Peptide	MW[a]	HBA[b]	HBD[c]	ROB[d]	CLP[e]	TPSA[f]	% ABS[g]	Solubility[h]	GI[i]	Pgp[j]	BBBP[k]	CYP1A2[l]	CYP2C19[m]	CYP2C9[n]	CYP2D6[o]	CYP3A4[p]	Toxicity[q]
LAF	349.42	5	4	11	0.67	121.52	67.0756	Highly soluble	High	No	No	No	No	No	No	No	non-toxic
LE	260.29	6	4	9	-0.63	129.72	64.2466	Highly soluble	High	No	No	No	No	No	No	No	non-toxic
LIV	343.46	5	4	12	0.93	121.52	67.0756	Very soluble	High	No	No	No	No	No	No	No	non-toxic
VLV	329.44	5	4	11	0.53	121.52	67.0756	Highly soluble	High	No	No	No	No	No	No	No	non-toxic
LLL	357.49	5	4	13	1.13	121.52	67.0756	Very soluble	High	No	No	No	No	No	No	No	non-toxic
GLF	335.4	5	4	11	0.36	121.52	67.0756	Highly soluble	High	No	No	No	No	No	No	No	non-toxic
LAL	315.41	5	4	11	0.41	121.52	67.0756	Highly soluble	High	No	No	No	No	No	No	No	non-toxic
AVL	301.38	5	4	10	-0.08	121.52	67.0756	Highly soluble	High	No	No	No	No	No	No	No	non-toxic
ALA	273.33	5	4	9	-0.61	121.52	67.0756	Highly soluble	High	No	No	No	No	No	No	No	non-toxic
VYV	379.45	6	5	11	0.5	141.75	60.09625	Very soluble	High	No	No	No	No	No	No	No	non-toxic
VLA	301.38	5	4	10	-0.44	121.52	67.0756	Highly soluble	High	No	No	No	No	No	No	No	non-toxic
GFL	335.4	5	4	11	0.51	121.52	67.0756	Very soluble	High	No	No	No	No	No	No	No	non-toxic
ALI	315.41	5	4	11	0.57	121.52	67.0756	Very soluble	High	No	No	No	No	No	No	No	non-toxic
VVG	273.33	5	4	9	-0.57	121.52	67.0756	Highly soluble	High	No	No	No	No	No	No	No	non-toxic
LLG	301.38	5	4	11	0.14	121.52	67.0756	Highly soluble	High	No	No	No	No	No	No	No	non-toxic
IVG	287.36	5	4	10	0	121.52	67.0756	Highly soluble	High	No	No	No	No	No	No	No	non-toxic
IIA	315.41	5	4	11	0.38	121.52	67.0756	Highly soluble	High	No	No	No	No	No	No	No	non-toxic
VAG	245.28	5	4	8	-1.16	121.52	67.0756	Highly soluble	High	No	No	No	No	No	No	No	non-toxic
LLA	315.41	5	4	11	0.41	121.52	67.0756	Highly soluble	High	No	No	No	No	No	No	No	non-toxic
YPG	335.36	6	4	8	-0.58	132.96	63.1288	Highly soluble	High	No	No	No	No	No	No	No	non-toxic
FQ	293.32	5	4	9	-0.63	135.51	62.24905	Highly soluble	High	No	No	No	No	No	No	No	non-toxic
APG	**243.26**	**5**	**3**	**6**	**-1.32**	**112.73**	**70.10815**	**Highly soluble**	**High**	**No**	**No**	**No**	**No**	**No**	**No**	**No**	**non-toxic**
TY	282.29	6	5	7	-0.97	132.88	63.1564	Highly soluble	High	No	No	No	No	No	No	No	non-toxic

PAY	349.38	6	5	9	-0.24	127.76	64.9228	Highly soluble	High	No	No	No	No	No	No	No	non-toxic
FA	236.27	4	3	6	-0.05	92.42	77.1151	Highly soluble	High	No	No	No	No	No	No	No	non-toxic
SL	218.25	5	4	7	-0.8	112.65	70.13575	Highly soluble	High	No	No	No	No	No	No	No	non-toxic
AL	202.25	4	3	6	-0.32	92.42	77.1151	Highly soluble	High	No	No	No	No	No	No	No	non-toxic
LA	202.25	4	3	6	-0.3	92.42	77.1151	Highly soluble	High	No	No	No	No	No	No	No	non-toxic
LT	232.28	5	4	7	-0.69	112.65	70.13575	Highly soluble	High	No	No	No	No	No	No	No	non-toxic
IV	230.3	4	3	7	0.21	92.42	77.1151	Highly soluble	High	No	No	No	No	No	No	No	non-toxic
II	244.33	4	3	8	0.54	92.42	77.1151	Highly soluble	High	No	No	No	No	No	No	No	non-toxic
PW	301.34	4	4	6	0.5	94.22	76.4941	Highly soluble	High	No	No	No	No	No	No	No	non-toxic
PPG	269.3	5	3	6	-0.92	98.74	74.9347	Highly soluble	High	No	No	No	No	No	No	No	non-toxic
WP	301.34	4	3	5	0.6	99.42	74.7001	Very soluble	High	No	No	No	No	No	No	No	non-toxic
SP	202.21	5	3	4	-1.55	103.86	73.1683	Highly soluble	High	No	No	No	No	No	No	No	non-toxic
MP	246.33	4	2	6	-0.24	108.93	71.41915	Highly soluble	High	No	No	No	No	No	No	No	non-toxic
PGP	253.3	4	2	6	-0.3	78.51	81.91405	Very soluble	High	No	No	No	No	No	No	No	non-toxic

[a] MW: Molecular weight; [b] HBA: H-bond acceptors; [c] HBD: H-bond donors; [d] ROB: Rotatable bonds; [e] CLP: Consensus Log P; [f] TPSA: Topological polar surface area; [g] % ABS: The percentage of absorption (% ABS) was calculated using TPSA by using formula %ABS=109- (0.345xTPSA); [h] Solubility: Water solubility was predicted by Ali model; [i] GI: Gastrointestinal absorption; [j] Pgp: P-glycoprotein substrate; [k] BBBP: Blood-brain barrier permeability; [l] CYP1A2: Cytochrome P450 1A2 inhibitor; [m] CYP2C19: Cytochrome P450 2C19 inhibitor; [n] CYP2C9: Cytochrome P450 2C9 inhibitor; [o] CYP2D6: Cytochrome P450 2D6 inhibitor; [p] CYP3A4: Cytochrome P450 3A4 inhibitor; [q] Toxicity: The ToxinPred (https://webs.iiitd.edu.in/raghava/toxinpred/index.html) was used to predict the toxicity of each peptide by selecting the SVM (Swiss-Prot)-based prediction model.

Table S10. The semi-flexible docking results of ACE receptor (rigid) with the different ligands (flexible)

No.	Ligand	-CDOCKER_ENERGY (kcal/mol)	Hydrogen bonding interaction											
			S1			S2					S1'	Zinc-binding domain		
			Ala354	Glu384	Tyr523	Gln281	His353	Lys511	His513	Tyr520	Glu162	His383	His387	Glu411
1	O_*Lisinopril*[a]	-	1	0	1	0	0	0	1	1	0	1	0	0
2	R_*Lisinopril*[b]	83.7945	1	1	1	0	1	1	2	1	1	0	0	0
3	LAF	96.3177	2	2	1	0	1	0	0	0	2	1	0	0
4	LE	95.7211	0	0	1	1	2	1	1	1	0	0	0	0
5	LIV	95.2431	2	1	1	0	1	0	1	0	0	1	0	0
6	VLV	95.0447	2	2	1	0	0	0	1	0	2	1	0	0
7	LLL	94.5727	1	1	1	0	1	0	2	0	0	1	0	0
8	GLF	94.2039	1	1	1	0	1	0	2	0	1	1	0	0
9	LAL	93.7997	2	2	1	0	1	0	0	0	1	1	0	0
10	AVL	92.8948	2	1	1	0	1	0	2	0	1	1	0	0
11	ALA	92.2416	2	1	1	0	2	0	1	0	1	1	0	0
12	VYV	92.1606	1	1	1	0	1	0	1	0	0	1	0	0

13	VLA	91.2098	2	2	1	0	1	0	0	0	2	0	0	0
14	GFL	90.8715	0	1	1	0	1	0	2	0	0	1	0	0
15	ALI	90.4492	2	1	1	0	0	0	1	0	2	1	0	0
16	VVG	90.3327	1	1	1	0	1	0	0	0	2	1	0	0
17	LLG	90.3278	1	1	1	0	1	0	1	0	0	1	0	0
18	IVG	90.2848	1	1	1	0	1	0	0	0	2	1	1	0
19	IIA	89.1223	1	1	1	0	1	0	2	0	0	1	0	0
20	VAG	87.1786	2	1	1	0	1	0	0	0	2	1	0	0
21	LLA	85.0248	1	1	1	0	1	0	2	0	0	1	0	0
22	YPG	83.7105	3	1	1	0	1	0	0	0	1	0	0	0
23	FQ	79.0369	1	1	2	0	0	0	0	0	0	1	0	0
24	APG	75.7973	3	1	1	0	1	0	0	0	2	0	0	0
25	TY	75.2897	1	1	1	0	0	0	1	0	0	1	0	0
26	PAY	73.1791	2	1	1	0	0	0	1	0	2	1	0	0
27	FA	72.9914	2	2	1	0	1	0	2	0	0	1	0	0

28	SL	71.834	2	1	1	0	0	0	1	0	0	1	0	0
29	AL	71.5918	1	2	1	0	0	0	1	0	0	1	0	0
30	LA	71.0061	1	2	1	0	0	0	1	0	0	1	0	0
31	LT	70.6531	3	1	1	0	1	0	1	0	0	0	0	0
32	IV	67.2541	2	1	1	0	1	0	1	0	0	0	0	0
33	II	66.8384	2	1	1	0	1	0	1	0	0	1	0	0
34	PW	56.7331	1	1	1	0	0	0	2	0	0	1	0	0
35	PPG	56.0776	1	1	1	0	1	0	1	0	1	1	1	0
36	WP	53.1217	2	1	2	0	0	0	1	0	0	1	1	0
37	SP	52.0736	2	1	2	0	0	0	1	0	0	1	1	0
38	MP	49.6497	1	1	2	0	0	0	1	0	0	1	1	0
39	PGP	44.3609	2	1	1	0	0	0	2	0	0	0	0	0

[a] O_*Lisinopril*: The interaction of *Lisinopril* with ACE was observed after the addition of hydrogen ions to the original crystal structure; [b] R_*Lisinopril*: Drug molecule-receptor interactions observed by re-docking of *Lisinopril* with ACE. *Lisinopril* was extracted from the original crystal structure.

Table S11 Interaction of ACE receptors with eight peptides

Ligand	-CDOCKER_ENERGY (kcal/mol)	Positive receptor-ligand interaction												Zn701 interaction
		S1			S2					S1'	Zinc-binding domain			
		Ala354	Glu384	Tyr523	Gln281	His353	Lys511	His513	Tyr520	Glu162	His383	His387	Glu411	
Lisinopril[a]	83.7945	1 Co-H[b]	1 Co-H	1 Co-H<br>1 PiC[c]	0	1 Co-H<br>1 AC[d]	1 SB-H[e]<br>(or AC)	1 Co-H<br>1 Ca-H[f]	1 Co-H	1 Co-H	1 PiC	0	0	1 AC<br>(or MA[g])
LAF	96.3177	2 Co-H	1 Co-H<br>1 Ca-H	1 Co-H	0	1 Co-H<br>1 PiA[h]	0	0	0	2 SB-H	1 Ca-H	0	0	1 AC<br>1 MA
LE	95.7211	0	0	1 Co-H	1 Co-H	1 Ca-H<br>1 SB-H<br>(or AC)	1 Co-H<br>1 AC	1 Ca-H	1 Co-H	0	1 PiA	0	0	1 AC<br>1 MA
LIV	95.2431	2 Co-H	1 Co-H	1 Co-H<br>1 PiA	0	1 Co-H<br>2 PiA	0	1 Ca-H<br>1 PiA	0	1 AC	1 Ca-H<br>2 PiA	0	0	1 AC
VLV	95.0447	2 Co-H	1 Co-H<br>1 Ca-H	1 Co-H	0	3 PiA	0	1 Ca-H<br>1 PiA	0	2 SB-H<br>(or AC)	1 Ca-H<br>1 PiA	0	0	1 AC
LLL	94.5727	1 Co-H	1 Co-H	1 Co-H	0	1 Co-H<br>2 PiA	0	1 Co-H<br>1 Ca-H	0	1 AC	1 Ca-H<br>1 PiA	0	0	1 AC
GLF	94.2039	1 Co-H	1 Co-H	1 Co-H	0	1 PiD-H[i]	0	1 Co-H<br>1 Ca-H	0	1 SB-H<br>(or AC)	1 Ca-H<br>1 PiA	0	0	1 AC
LAL	93.7997	2 Co-H	1 Co-H<br>1 Ca-H	1 Co-H	0	1 Co-H	0	1 PiA	0	1 SB-H<br>(or AC)	1 Ca-H	0	0	1 AC<br>(or MA)
AVL	92.8948	2 Co-H	1 Co-H	1 Co-H<br>1 PiA	0	1 Co-H<br>1 PiA	0	1 Co-H<br>1 Ca-H	0	1 SB-H<br>(or AC)	1 Ca-H<br>1 PiA	0	0	1 AC

[a] *Lisinopril*: Drug molecule-receptor interactions observed by re-docking of *Lisinopril* with ACE. *Lisinopril* was extracted from the original crystal structure. [b] Co-H: Conventional Hydrogen Bond. [c] PiC: Pi-Cation. [d] AC: Attractive Charge. [e] SB-H: Salt Bridge. [f] Ca-H: Carbon

Hydrogen Bond. [g] MA: Metal-Acceptor. [h] PiA: Pi-Alkyl. [i] PiD-H: Pi-Donor Hydrogen Bond. The abbreviation suffix "-H" stands for hydrogen bond, otherwise it means non-hydrogen bond.

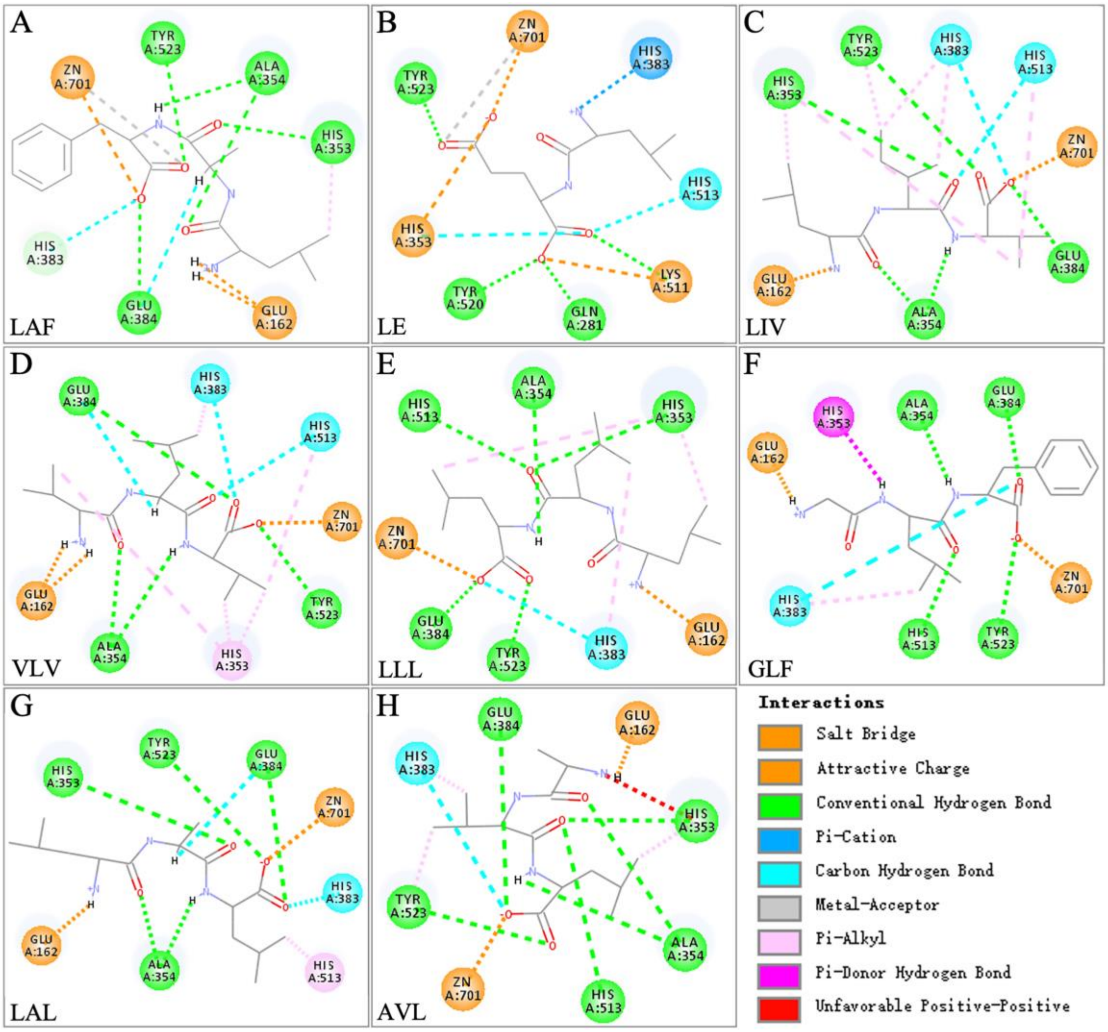

Figure S1. 2D-diagram of the interaction between eight pre-MBPs and ACE. The non-bonded interactions of (A) LAF, (B) LE, (C) LIV, (D) VLV, (E) LLL, (F) GLF, (G) LAL, and (H) AVL with ACE, respectively

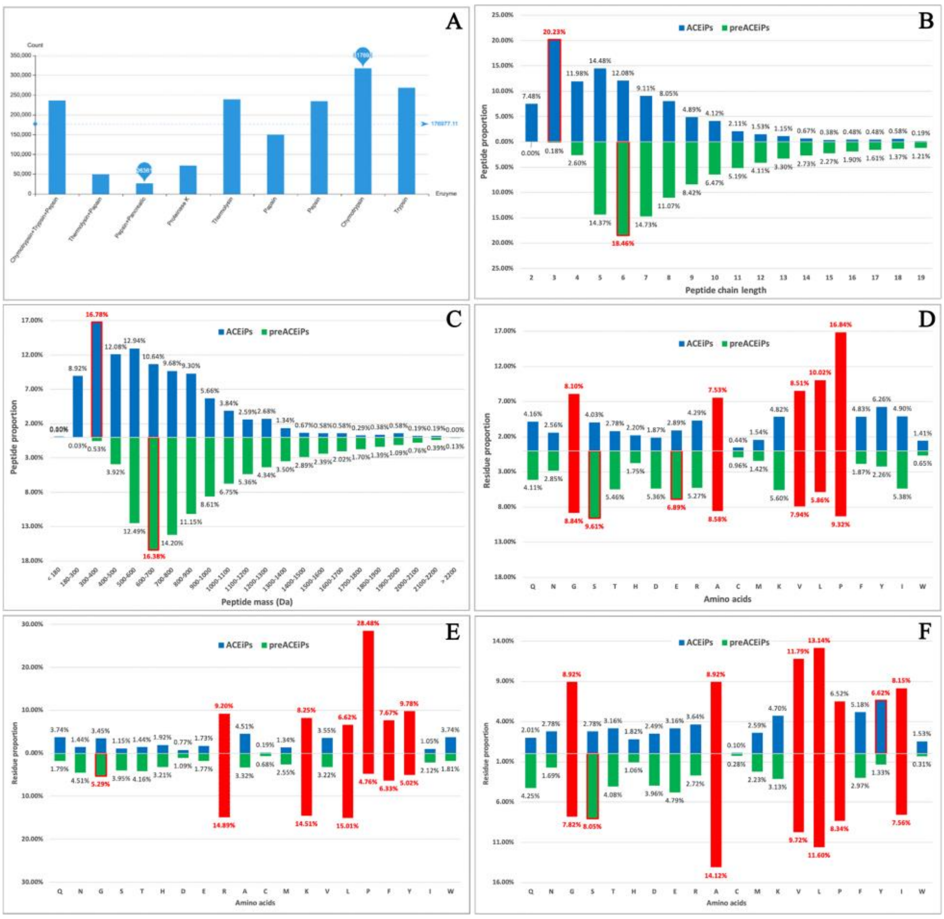

Figure S2. Hydrolyzed peptide prediction (1,320,635 unique sequences, probability ≥ 0.5) and their sequence feature comparison with experimental ACEiPs (1043 unique sequences). (A) Quantitative statistics of the predicted ACEiPs obtained by 9 different enzymatic hydrolysis methods (2 ≤ length ≤ 19); (B) Length distribution: The experimental ACEiPs are concentrated in tripeptides to octapeptides (mainly tripeptides and pentapeptides), whereas the predicted ACEiPs were mainly concentrated in pentapeptides to octapeptides, with the most for hexapeptides; (C) Mass distribution: The experimental ACEiPs and preACEiPs are mainly concentrated in < 1000 Da, and their largest sample sizes are concentrated in 300-400 Da and 600-700 Da, respectively; (D) Residue composition: Experimental and predicted ACEiPs are all mainly composed

of hydrophobic amino acids (Pro, Leu, Val, Gly, and Ala); (E) C-terminal residue composition: Experimental and predicted ACEiPs have similar C-terminal residue composition, including hydrophobic (Pro, Phe, and Leu), positively charged (Arg and Lys), and bulky aromatic (Tyr) amino acids; (F) N-terminal residue composition: Experimental and predicted ACEiPs have similar N-terminal hydrophobic amino acid composition (Leu, Val, Ala, Gly, Ile, and Pro).

References

Cocchi, M., & Johansson, E. (1993). Amino Acids Characterization by GRID and Multivariate Data Analysis. *Quantitative Structure-Activity Relationships, 12*(1), 1-8.

Collantes, E. R., & Dunn, W. J., 3rd. (1995). Amino acid side chain descriptors for quantitative structure-activity relationship studies of peptide analogues. *J Med Chem, 38*(14), 2705-2713.

Georgiev, A. G. (2009). Interpretable numerical descriptors of amino acid space. *Journal of Computational Biology, 16*(5), 703-723.

Hellberg, S., Sjostrom, M., Skagerberg, B., & Wold, S. (1987). Peptide quantitative structure-activity relationships, a multivariate approach. *J Med Chem, 30*(7), 1126-1135.

Hemmateenejad, B., Yousefinejad, S., & Mehdipour, A. R. (2011). Novel amino acids indices based on quantum topological molecular similarity and their application to QSAR study of peptides. *Amino Acids, 40*(4), 1169-1183.

Henikoff, S., & Henikoff, J. G. (1992). Amino acid substitution matrices from protein blocks. *Proceedings of the National Academy of Sciences, 89*(22), 10915-10919.

Hernández-Ledesma, B., del Mar Contreras, M., & Recio, I. (2011). Antihypertensive peptides: Production, bioavailability and incorporation into foods. *Advances in Colloid and Interface Science, 165*(1), 23-35.

Jurtz, V. I., Johansen, A. R., Nielsen, M., Almagro Armenteros, J. J., Nielsen, H., Sønderby, C. K., Winther, O., & Sønderby, S. K. (2017). An introduction to deep learning on biological sequence data: examples and solutions. *Bioinformatics, 33*(22), 3685-3690.

Liang, G.-Z., Shu, M., & Li, S.-S. Z. (2008). A new set of amino acid descriptors for the development of quantitative sequence-activity modelings of HLA-A*0201 restrictive CTL epitopes. *J CHIN CHEM SOC-TAIP, 55*(5), 1178-1185.

Liang, G., & Li, Z. (2007). Factor analysis scale of generalized amino acid Information as the source of a new set of descriptors for elucidating the structure and activity relationships of cationic antimicrobial peptides. *QSAR & Combinatorial Science, 26*(6), 754-763.

Liang, G., Liu, Y., Shi, B., Zhao, J., & Zheng, J. (2013). An index for characterization of natural and non-natural amino acids for peptidomimetics. *PLoS One, 8*(7), e67844.

Lin, Z. H., Long, H. X., Bo, Z., Wang, Y. Q., & Wu, Y. Z. (2008). New descriptors of amino acids and their application to peptide QSAR study. *Peptides, 29*(10), 1798-1805.

Mei, H., Liao, Z. H., Zhou, Y., & Li, S. Z. (2005). A new set of amino acid descriptors and its application in peptide QSARs. *Biopolymers, 80*(6), 775-786.

Sagardia, I., Roa-Ureta, R. H., & Bald, C. (2013). A new QSAR model, for angiotensin I-converting enzyme inhibitory oligopeptides. *Food Chemistry, 136*(3-4), 1370-1376.

Shu, M., Cheng, X., Zhang, Y., Wang, Y., Lin, Y., Wang, L., & Lin, Z. (2011). Predicting the activity of ACE inhibitory peptides with a novel mode of pseudo amino acid composition. *Protein Pept Lett, 18*(12), 1233-1243.

Shu, M., Mei, H., Yang, S., Liao, L., & Li, Z. (2009). Structural parameter characterization and bioactivity simulation based on peptide sequence. *QSAR & Combinatorial Science, 28*(1), 27-35.

Tian, F., Yang, L., Lv, F., Yang, Q., & Zhou, P. (2009). In silico quantitative prediction of peptides binding affinity to human MHC molecule: an intuitive quantitative structure-activity relationship approach. *Amino Acids, 36*(3), 535-554.

Tian, F., Zhou, P., & Li, Z. (2007). T-scale as a novel vector of topological descriptors for amino acids and its application in QSARs of peptides. *Journal of Molecular Structure, 830*(1-3), 106-115.

Tong, J., Che, T., Li, Y., Wang, P., Xu, X., & Chen, Y. (2011). A descriptor of amino acids: SVRG and its application to peptide quantitative structure-activity relationship. *SAR and QSAR in Environmental Research, 22*(5-6), 611-620.

Tong, J., Chen, Y., Liu, S., Che, T., & Xu, X. (2012). A descriptor of amino acids SVWG and its applications in peptide QSAR. *J CHEMOMETR, 26*(10), 549-555.

Tong, J., Li, L., Bai, M., & Li, K. (2017). A new descriptor of amino acids-SVGER and its applications in peptide QSAR. *Molecular Informatics, 36*(5-6), 1501023.

Tong, J., Liu, S., Zhou, P., Wu, B., & Li, Z. (2008). A novel descriptor of amino acids and its application in peptide QSAR. *J Theor Biol, 253*(1), 90-97.

van Westen, G. J., Swier, R. F., Wegner, J. K., Ijzerman, A. P., van Vlijmen, H. W., & Bender, A. (2013). Benchmarking of protein descriptor sets in proteochemometric modeling (part 1): comparative study of 13 amino acid descriptor sets. *Journal of Cheminformatics, 5*(1), 41.

Wu, J., Aluko, R. E., & Nakai, S. (2006). Structural requirements of Angiotensin I-converting enzyme inhibitory peptides: quantitative structure-activity relationship study of di- and tripeptides. *Journal of agricultural and food chemistry, 54*(3), 732-738.

Xiang, L., Qiu, Z., Zhao, R., Zheng, Z., & Qiao, X. (2021). Advancement and prospects of production, transport, functional activity and structure-activity relationship of food-derived angiotensin converting enzyme (ACE) inhibitory peptides. *Critical Reviews in Food Science and Nutrition*, 1-27.

Yang, L., Shu, M., Ma, K., Mei, H., Jiang, Y., & Li, Z. (2010). ST-scale as a novel amino acid descriptor and its application in QSAM of peptides and analogues. *Amino Acids, 38*(3), 805-816.

Zaliani, A., & Gancia, E. (1999). MS-WHIM scores for amino acids: A new 3D-description for peptide QSAR and QSPR studies. *Journal of Chemical Information and Computer Sciences, 39*(3), 525-533.
